# Thermodynamic and Ultrasonic Properties of Ascorbic Acid in Aqueous Protic Ionic Liquid Solutions

**DOI:** 10.1371/journal.pone.0126091

**Published:** 2015-05-26

**Authors:** Vickramjeet Singh, Gyanendra Sharma, Ramesh L. Gardas

**Affiliations:** Department of Chemistry, Indian Institute of Technology Madras, Chennai—600 036, India; King's College London, UNITED KINGDOM

## Abstract

In this work, we report the thermodynamic and ultrasonic properties of ascorbic acid (vitamin C) in water and in presence of newly synthesized ammonium based protic ionic liquid (diethylethanolammonium propionate) as a function of concentration and temperature. Apparent molar volume and apparent molar isentropic compression, which characterize the solvation state of ascorbic acid (AA) in presence of protic ionic liquid (PIL) has been determined from precise density and speed of sound measurements at temperatures (293.15 to 328.15) K with 5 K interval. The strength of molecular interactions prevailing in ternary solutions has been discussed on the basis of infinite dilution partial molar volume and partial molar isentropic compression, corresponding volume of transfer and interaction coefficients. Result has been discussed in terms of solute-solute and solute-solvent interactions occurring between ascorbic acid and PIL in ternary solutions (AA + water + PIL).

## Introduction

Ionic liquids (ILs) are molten salts with melting point below 100°C. ILs have unique physicochemical properties such as broad liquid temperature range, negligible vapor pressure, wide electrochemical window,high thermal stability, and high specific solvent abilities [1–5]. The thermo-physical properties of ILs can be tuned by appropriate selection of cation and anion, as a result ILs can be made biocompatible and these are found to be very attractive in various analytical applications, particularly, in the fabrication of various modified electrodeswhichcan be used to extract chemicals or compounds (synthetic colors) from food samples. Reports are available [6–12]were ILs have been used for the extraction of Sudan and Para Red dyes from chilli powder employinghigh performance liquid chromatography (HPLC), thus showing potential application of ILs in food industries [10–11,13–15].

Chailapakul et al. [16] have used IL based carbon electrode for the analysis of sudan I, sudan II, sudan III and sudan IV. Recently, a novel carbon composite electrode of an IL (n-octylpyridinum hexafluorophosphate) and single-walled carbon nanotube (SWCNT) was designed to determine the levels of ascorbic acid from food samples [11]. Ionic liquids have also been proposed as an effective compound to be used in the formation of aqueous biphasic system (ABS) [17–18]. ABS are considered as an alternative to liquid-liquid extraction techniques, as ABS are being used as powerful technique in bioseparation processes, purification, extraction and enrichment [18–19]. ABS with ILs in combination with salts, amino acids, polyhydroxy compounds (saccharides), and polymers has been reported [18–24]. More recently, it has been reported [25] that the ABS employing ILs with various solutes (saccharides, amino acids, vitamins, etc) requires reliable and systematic thermodynamic data. The acquaintance of thermodynamics of ILs in aqueous solutions with various biomolecules (saccharides, amino acid, etc) is of crucial importance to improve the process design and to understand the molecular interactions between ILs and biomolecules, thus serving with the design of ABS systems.

Furthermore, the increased utilization of ILs for various applications (chemical or separations processes) requires accurate determination of thermodynamic data [25]. The nature and strength of interactions between AA and various co-solutes (electrolytes, non electrolytes, surfactants etc.) have been studied [26–31] by evaluating the thermodynamic properties of ternary systems (AA + water + co-solutes). These thermodynamic properties are useful in characterizing the solvation behaviour of vitamins and to further understand solute-solute and solute-solvent interactions [32–33]. Ascorbic acid (vitamin C) one of the most important vitamin for human health and nutrition, is found in fruits and vegetables [34–39]. It is a sugar acid, having antioxidant properties and can prevent or treat common cold and scurvy. It also acts as a cofactor and thus maintain activity of various enzymes [26–27,32,40–41]. The degradation of AA is very important and is considered to be the major cause of color and quality change during storage or processing of food materials [40, 42].

However, to the best of our knowledge there is no report on thermodynamic and ultrasonic properties of AA in aqueous solutions of ammonium based protic ionic liquid (PIL) as a function of concentration and temperature. So, in order to understand the molecular interactions occurring between AA and PIL, we report herein the volumetric propertiesof AA in aqueous solutions of newly synthesized protic ionic liquid i.e. diethylethanolammonium propionate([DEEA][Pro]) at different temperatures (293.15 to 328.15) K. Various parameters such as partial molar volumes and isentropic compression, transfer volumes, interaction coefficients, and thermal expansion coefficients have been evaluated and discussed in terms of solute-solute and solute-cosolute interactions.

## Materials and Methods

### 2.1 Materials

Ascorbic acid (mass fraction purity; 0.99) was purchased from Sisco Research Laboratory Pvt. Ltd. India, N,N-diethylethanol amine (0.99), propanoic acid (0.99)and methanol (0.99) were purchased from Sigma Aldrich. AA was used after drying in a vacuum desiccator (over anhydrous CaCl_2_) for 48 h at room temperature and all the other chemicals were used without further purification.

### 2.2. Synthesis and characterization of ([DEEA][Pro])

PIL, ([DEEA][Pro]) was synthesized by exothermic neutralization of bronsted acid (propanoic acid) by base (N,N- diethylethanol amine). To10 ml of methanol, N,N- diethylethanol amine (0.1mol) was added in round bottom flask and this mixture was kept in ice bath for few minutes. Further, propanoic acid (0.11mol) was added slowly and drop wise (by using dropping funnel) to the above reaction mixture. Addition of acid was completed in 2 hrs at a temperature below 5°C and then reaction mixture was stirred continuously for 24 hrs at room temperature. The excess amount of starting material and solvent were removed by putting the reaction mixture into rotavapor for 4 hrs. The resultant product (PIL) was dried at room temperature under high vacuum for 36 hrs, in order to remove moisture and excess of amineand further IL was kept in N_2_ atmosphere.

Synthesized PIL was characterized by ^1^H NMR, ^13^C NMR (Bruker Avance 400 MHz) and FTIR (JASKO FT/IR-4100) spectroscopictechniques. NMR was recorded in CDCl_3_. ^1^H NMR of [DEEA][Pro], δ = 4.725ppm (broad, 2H, OH and NH^+^), δ = 3.863ppm (t, 2H), δ = 3.049ppm (q, 3H), δ = 2.972ppm (t, 2H), δ = 2.287ppm (q, 2H), δ = 1.250ppm (t, 6H) and δ = 1.119ppm (t, 3H). IR was recorded using KBr disk, the JASKO FT/IR-4100 spectrometer has a maximum resolution of 0.9 cm^-1^ and signal to noise ratio of 22000:1. The IR broad band appeared in range of 3400–2800 cm^-1^ correspondsto the characteristic ammonium peak, ν(N-H) and ν(O-H) stretching vibration. The broad band centered around 1600 cm^−1^corresponds to the characteristic carbonyl, ν(C = O) stretching and δ(N-H) plane bending, vibrations. The Karl Fischer titrator from Analab (Micro Aqua Cal 100) was used to measure the water content. This instrument operates on conductometric titration principle using dual platinum electrodes that permits detection of water content from less than 10 ppm to 100%. The water content in the synthesized [DEEA][Pro] was ≈ 7000 ppm. The amount of water present in PIL has been taken into account for the molality correction of stock solutions (water + PIL).

### 2.3 Density and speed of sound

The vibrating-tube digital density meter and sound velocity analyzer (Anton Paar, DSA 5000M) was used to measure simultaneously the densities, *ρ* and speeds of sound, *u* of AA in water and in *m*_B_ (molality of PIL) = (0.10, 0.15, 0.20, and 0.25) mol·kg^-1^ aqueous solutions of [DEEA][Pro] at temperatures, *T* = (293.15, 298.15, 303.15, 308.15, 313.15, 318.15, 323.15 and 328.15) K and at atmospheric pressure. The instrument is equipped with a density cell and a sound velocity cell, which are temperature controlled by a built-in Peltier thermostat (PT-100) with an accuracy of ±0.01 K. It can measure the density in the range of (0 to 3000) kg·m^-3^ and speed of sound from (1000 to 2000) m·s^-1^. At regular intervals, instrument was calibrated at atmospheric pressure with dry air and deionized, double distilled, and freshly degassed water according to the procedure mentioned in the instrument manual. The uncertainties in the measurement of density and speed of sound were ± 7×10ˉ^3^ kg∙mˉ^3^ and ±0.5 m∙sˉ^1^, respectively. The solutions were made fresh in Millipore quality freshly degassed water on mass basis in air tight glass vials by using Sartorius balance (Model CPA225D) having a precision of ±0.01 mg. The uncertainty in molality was ± 1.03·10^-5^ mol·kg^-1^.

## Results and Discussion

### 3.1 Apparent molar volume and apparent molar isentropic compression

The understanding of molecular interaction between a solute and solvent (water) andthe packing efficiency of solute within the structure of water has been studied in aqueous [43–44] and mixed aqueous solutions [3,45–47]. The packing efficiency of a solute which is governed by solute-solvent interactions can be measured by employing apparent molar volume. Apparent molar volume is smaller for heavily hydrated molecules as compared to those which are weakly hydrated, and this may be due to greater interaction of solute molecules with water [48]. The solvation behaviour of a solute has been studied by two most important parameters i.e.apparent molar volume, *V*_2,ϕ_ and apparent molar isentropic compression, *K*_s,2, ϕ._ In this study, the apparent molar volume, *V*_2, ϕ_ and apparent molar isentropic compression, *K*_s,2, ϕ_ of AAin water and in *m*_B_ = (0.10, 0.15, 0.20, and 0.25) mol·kg^-1^ aqueous solutions of [DEEA][Pro] at different temperatures (Table 1) were determined form density and speed of sound data (Table 1) by using the following Eqs (1) and (2):

V2,ϕ=⌈Mρ⌉−[(ρ–ρo)/(m⋅ρ⋅ρo)] (1)

Ks,2,ϕ=⌈κs .Mρ⌉−[(κso⋅ρ –κs ρo)/(m⋅ρ⋅ρo)] (2)

where *M* and *m* are respectively, the molar mass and molality of AA; *ρ* and *ρ*_o_ are the densities of solution and solvent (water or water + [DEEA][Pro]), *κ*_s_and*κ*_s_° are the isentropic compressibilities of solution and solvent, respectively.

**Table 1:** The densities, *ρ*, apparent molar volumes, *V*_2, ϕ_, speeds of sound, *u* and apparent molar isentropic compression, K_s,2, ϕ°_ of ascorbic acid in water and in aqueous [DEEA][Pro] solutions at temperatures, *T* = (293.15 to 328.15) K and at ambient pressure.

*T/K*	^a^ *m*_B_	^b^ *m*	*ρ*·10^−3^	*V*_*2*,*ϕ*_·10^6^	*u*	10^15^·*K*_s,2, ϕ_
	mol·kg^−1^	mol·kg^−1^	kg·m^−3^	m^3^·mol^−1^	m·s^−1^	m^3^·mol^−1^·Pa^−1^
293.15	*0*.*0*		(^c^ *ρ*_o_ = 0.998206)		(^d^ *u*_o_ = 1482.84)	
		0.06924	1.003091	105.13	1485.32	-12.16
		0.10269	1.005422	105.16	1486.84	-12.12
		0.15500	1.009032	105.21	1489.23	-12.06
		0.19851	1.012001	105.25	1491.23	-12.00
		0.25161	1.015588	105.28	1493.69	-11.95
		0.27597	1.017216	105.31	1494.82	-11.91
		0.33464	1.021103	105.36	1497.56	-11.83
298.15			(^c^ *ρ*_o_ = 0.997049)		(^d^ *u*_o_ = 1496.85)	
		0.06924	1.001890	105.81	1499.06	-8.59
		0.10269	1.004200	105.84	1500.43	-8.54
		0.15500	1.007777	105.89	1502.58	-8.45
		0.19851	1.010721	105.92	1504.39	-8.43
		0.25161	1.014275	105.95	1506.60	-8.36
		0.27597	1.015888	105.98	1507.61	-8.30
		0.33464	1.019740	106.03	1510.07	-8.21
303.15			(^c^ *ρ*_o_ = 0.995660)		(^d^ *u*_o_ = 1509.25)	
		0.06924	1.000470	106.31	1511.65	-5.50
		0.10269	1.002766	106.34	1512.88	-5.47
		0.15500	1.006321	106.38	1514.82	-5.43
		0.19851	1.009246	106.41	1516.44	-5.37
		0.25161	1.012775	106.45	1518.43	-5.31
		0.27597	1.014375	106.49	1519.35	-5.27
		0.33464	1.018204	106.53	1521.56	-5.18
308.15			(^c^ *ρ*_o_ = 0.994045)		(^d^ *u*_o_ = 1519.82)	
		0.06924	0.998811	107.01	1521.41	-4.01
		0.10269	1.001083	107.07	1522.59	-3.95
		0.15500	1.004602	107.12	1524.45	-3.89
		0.19851	1.007488	107.20	1526.02	-3.84
		0.25161	1.010983	107.23	1527.94	-3.80
		0.27597	1.012565	107.27	1528.82	-3.75
		0.33464	1.016344	107.34	1530.96	-3.67
313.15			(^c^ *ρ*_o_ = 0.992228)		(^d^ *u*_o_ = 1528.89)	
		0.06924	0.996961	107.57	1531.16	0.01
		0.10269	0.999216	107.63	1532.14	0.03
		0.15500	1.002713	107.66	1533.67	0.09
		0.19851	1.005588	107.70	1534.95	0.15
		0.25161	1.009056	107.75	1536.53	0.21
		0.27597	1.010625	107.80	1537.27	0.24
		0.33464	1.014388	107.84	1539.04	0.29
318.15			(^c^ *ρ*_o_ = 0.990223)		(^d^ *u*_o_ = 1536.56)	
		0.06924	0.994936	107.94	1537.95	1.13
		0.10269	0.997180	108.02	1538.87	1.19
		0.15500	1.000658	108.07	1540.32	1.24
		0.19851	1.003515	108.13	1541.54	1.29
		0.25161	1.006965	108.18	1543.03	1.36
		0.27597	1.008523	108.24	1543.72	1.42
		0.33464	1.012253	108.32	1545.39	1.51
323.15			(^c^ *ρ*_o_ = 0.9888030)		(^d^ *u*_o_ = 1542.73)	
		0.06924	0.992708	108.54	1544.94	2.56
		0.10269	0.994939	108.59	1545.79	2.63
		0.15500	0.998391	108.65	1547.14	2.67
		0.19851	1.001229	108.70	1548.27	2.73
		0.25161	1.004643	108.80	1549.67	2.80
		0.27597	1.006197	108.83	1550.32	2.82
		0.33464	1.009899	108.91	1551.88	2.91
328.15			(^c^ *ρ*_o_ = 0.985690)		(^d^ *u*_o_ = 1547.61)	
		0.06924	0.990343	109.00	1548.43	4.07
		0.10269	0.992562	109.05	1549.21	4.09
		0.15500	0.995996	109.11	1550.44	4.14
		0.19851	0.998819	109.16	1551.46	4.22
		0.25161	1.002229	109.20	1552.73	4.26
		0.27597	1.003773	109.24	1553.32	4.29
		0.33464	1.007462	109.31	1554.72	4.40
293.15	*0*.*10*		(^c^ *ρ*_o_ = 0.999919)		(^d^ *u*_o_ = 1499.13)	
		0.03740	1.002499	106.86	1500.41	-3.60
		0.06114	1.004132	106.78	1501.22	-3.58
		0.12575	1.008552	106.56	1503.40	-3.48
		0.14367	1.009777	106.47	1503.99	-3.44
		0.21169	1.014398	106.20	1506.22	-3.34
		0.27537	1.018711	105.90	1508.27	-3.30
		0.32896	1.022297	105.74	1510.00	-3.24
298.15			(^c^ *ρ*_o_ = 0.998732)		(^d^ *u*_o_ = 1512.18)	
		0.03740	1.001277	107.85	1513.00	1.08
		0.06114	1.002885	107.80	1513.66	1.12
		0.12575	1.007240	107.61	1515.44	1.17
		0.14367	1.008456	107.45	1515.90	1.21
		0.21169	1.013010	107.20	1517.71	1.28
		0.27537	1.017251	106.95	1519.35	1.36
		0.32896	1.020816	106.69	1520.68	1.43
			(^c^ *ρ*_o_ = 0.997302)		(^d^ *u*_o_ = 1523.70)	
303.15		0.03740	0.999822	108.58	1524.37	4.49
		0.06114	1.001422	108.41	1524.89	4.62
		0.12575	1.005741	108.22	1526.34	4.62
		0.14367	1.006948	108.06	1526.70	4.69
		0.21169	1.011488	107.70	1528.12	4.74
		0.27537	1.015719	107.38	1529.39	4.82
		0.32896	1.019256	107.15	1530.44	4.87
			(^c^ *ρ*_o_ = 0.995657)		(^d^ *u*_o_ = 1523.36)	
308.15		0.03740	0.998153	109.31	1534.45	6.17
		0.06114	0.999738	109.12	1534.93	6.21
		0.12575	1.004054	108.62	1536.17	6.31
		0.14367	1.005268	108.37	1536.48	6.31
		0.21169	1.009779	108.07	1537.72	6.41
		0.27537	1.014027	107.62	1538.78	6.46
		0.32896	1.017589	107.28	1539.62	6.53
			(^c^ *ρ*_o_ = 0.993814)		(^d^ *u*_o_ = 1542.00)	
313.15		0.03740	0.996285	110.07	1542.85	10.53
		0.06114	0.997860	109.77	1543.15	10.60
		0.12575	1.002098	109.61	1543.99	10.69
		0.14367	1.003299	109.34	1544.16	10.74
		0.21169	1.007759	109.00	1544.92	10.85
		0.27537	1.011956	108.54	1545.51	10.95
		0.32896	1.015446	108.29	1546.01	11.03
318.15			(^c^ *ρ*_o_ = 0.991786)		(^d^ *u*_o_ = 1548.91)	
		0.03740	0.994243	110.54	1549.79	12.76
		0.06114	0.995806	110.30	1550.01	12.80
		0.12575	1.000031	110.02	1550.59	12.91
		0.14367	1.001225	109.75	1550.69	12.98
		0.21169	1.005670	109.38	1551.20	13.03
		0.27537	1.009854	108.90	1551.56	13.10
		0.32896	1.013333	108.64	1551.86	13.17
323.15			(^c^ *ρ*_o_ = 0.989577)		(^d^ *u*_o_ = 1554.41)	
		0.03740	0.992010	111.27	1555.14	14.41
		0.06114	0.993558	111.04	1555.31	14.41
		0.12575	0.997740	110.78	1555.76	14.47
		0.14367	0.998923	110.51	1555.83	14.51
		0.21169	1.003326	110.13	1556.19	14.58
		0.27537	1.007422	109.83	1556.49	14.64
		0.32896	1.010879	109.50	1556.67	14.70
328.15			(^c^ *ρ*_o_ = 0.987199)		(^d^ *u*_o_ = 1558.62)	
		0.03740	0.989618	111.77	1558.77	16.16
		0.06114	0.991153	111.61	1558.88	16.14
		0.12575	0.995303	111.37	1559.15	16.25
		0.14367	0.996478	111.10	1559.17	16.29
		0.21169	1.000851	110.71	1559.35	16.33
		0.27537	1.004965	110.23	1559.41	16.39
		0.32896	1.008382	109.98	1559.46	16.46
			(^c^ *ρ*_o_ = 1.000704)		(^d^ *u*_o_ = 1506.73)	
293.15	*0*.*15*	0.05512	1.004497	106.88	1508.81	-2.16
		0.07286	1.005719	106.74	1509.37	-2.14
		0.11890	1.008869	106.56	1510.83	-2.10
		0.22113	1.015818	106.15	1514.00	-2.00
		0.27993	1.019776	105.95	1515.80	-1.95
		0.40125	1.027840	105.61	1519.50	-1.87
		0.45594	1.031449	105.43	1521.12	-1.83
			(^c^ *ρ*_o_ = 0.999489)		(^d^ *u*_o_ = 1519.38)	
298.15		0.05512	1.003234	107.80	1521.56	1.87
		0.07286	1.004440	107.66	1522.02	1.91
		0.11890	1.007543	107.55	1523.22	2.00
		0.22113	1.014361	107.30	1525.89	2.04
		0.27993	1.018234	107.18	1527.39	2.13
		0.40125	1.026161	106.83	1530.44	2.20
		0.45594	1.029681	106.71	1531.79	2.26
			(^c^ *ρ*_o_ = 0.998041)		(^d^ *u*_o_ = 1530.40)	
303.15		0.05512	1.001743	108.64	1531.25	5.30
		0.07286	1.002929	108.59	1531.63	5.35
		0.11890	1.006008	108.34	1532.60	5.36
		0.22113	1.012733	108.18	1534.78	5.44
		0.27993	1.016570	108.02	1535.99	5.49
		0.40125	1.024365	107.77	1538.48	5.60
		0.45594	1.027839	107.65	1539.59	5.63
			(^c^ *ρ*_o_ = 0.996383)		(^d^ *u*_o_ = 1539.88)	
308.15		0.05512	1.000044	109.47	1541.42	7.13
		0.07286	1.001223	109.33	1541.76	7.10
		0.11890	1.004252	109.24	1542.64	7.18
		0.22113	1.010912	109.00	1544.58	7.23
		0.27993	1.014702	108.85	1545.66	7.29
		0.40125	1.022405	108.61	1547.88	7.39
		0.45594	1.025833	108.50	1548.87	7.42
			(^c^ *ρ*_o_ = 0.994528)		(^d^ *u*_o_ = 1544.26)	
313.15		0.05512	0.998152	110.22	1544.30	11.42
		0.07286	0.999318	110.10	1544.51	11.46
		0.11890	1.002324	109.95	1545.07	11.47
		0.22113	1.008913	109.74	1546.29	11.56
		0.27993	1.012683	109.53	1546.93	11.62
		0.40125	1.020327	109.26	1548.29	11.70
		0.45594	1.023730	109.14	1548.86	11.76
318.15			(^c^ *ρ*_o_ = 0.992490)		(^d^ *u*_o_ = 1547.14)	
		0.05512	0.996079	110.96	1547.55	13.53
		0.07286	0.997233	110.85	1547.71	13.55
		0.11890	1.000207	110.71	1548.13	13.58
		0.22113	1.006709	110.60	1549.07	13.69
		0.27993	1.010407	110.49	1549.59	13.74
		0.40125	1.017946	110.23	1550.60	13.85
		0.45594	1.021312	110.09	1551.02	13.90
323.15			(^c^ *ρ*_o_ = 0.990281)		(^d^ *u*_o_ = 1559.57)	
		0.05512	0.993841	111.59	1560.29	15.10
		0.07286	0.994987	111.47	1560.40	15.14
		0.11890	0.997932	111.38	1560.71	15.17
		0.22113	1.004384	111.24	1561.39	15.25
		0.27993	1.008079	111.03	1561.73	15.28
		0.40125	1.015571	110.76	1562.44	15.36
		0.45594	1.018910	110.63	1562.72	15.41
328.15			(^c^ *ρ*_o_ = 0.987911)		(^d^ *u*_o_ = 1563.51)	
		0.05512	0.991440	112.29	1563.86	17.26
		0.07286	0.992572	112.21	1563.91	17.34
		0.11890	0.995490	112.11	1564.07	17.35
		0.22113	1.001870	112.02	1564.43	17.43
		0.27993	1.005509	111.88	1564.60	17.48
		0.40125	1.012869	111.73	1564.98	17.58
		0.45594	1.016151	111.63	1565.14	17.61
			(^c^ *ρ*_o_ = 1.001486)		(^d^ *u*_o_ = 1514.29)	
293.15	*0*.*20*	0.05334	1.005107	107.79	1516.85	-1.75
		0.10306	1.008484	107.42	1518.46	-1.71
		0.11587	1.009365	107.23	1518.85	-1.70
		0.16129	1.012422	107.10	1520.30	-1.62
		0.28817	1.020918	106.57	1524.25	-1.52
		0.31479	1.022701	106.42	1525.03	-1.47
		0.50949	1.035583	105.55	1530.65	-1.26
			(^c^ *ρ*_o_ = 1.000251)		(^d^ *u*_o_ = 1526.52)	
298.15		0.05334	1.003835	108.53	1528.04	2.20
		0.10306	1.007160	108.33	1529.38	2.27
		0.11587	1.008023	108.20	1529.70	2.32
		0.16129	1.011035	108.09	1530.91	2.36
		0.28817	1.019377	107.69	1534.20	2.47
		0.31479	1.021157	107.46	1534.80	2.51
		0.50949	1.033766	106.76	1539.48	2.68
			(^c^ *ρ*_o_ = 0.998785)		(^d^ *u*_o_ = 1537.14)	
303.15		0.05334	1.002327	109.39	1537.85	5.87
		0.10306	1.005613	109.18	1538.93	5.88
		0.11587	1.006469	109.03	1539.18	5.91
		0.16129	1.009459	108.84	1540.12	5.98
		0.28817	1.017700	108.49	1542.72	6.10
		0.31479	1.019446	108.31	1543.19	6.14
		0.50949	1.031941	107.54	1546.74	6.32
			(^c^ *ρ*_o_ = 0.997109)		(^d^ *u*_o_ = 1546.21)	
308.15		0.05334	1.000586	110.15	1548.20	7.74
		0.10306	1.003800	109.87	1549.21	7.76
		0.11587	1.004637	109.73	1549.44	7.81
		0.16129	1.007606	109.55	1550.26	7.83
		0.28817	1.015778	109.08	1552.51	7.96
		0.31479	1.017529	108.89	1552.88	8.00
		0.50949	1.030001	107.96	1555.78	8.17
313.15			(^c^ *ρ*_o_ = 0.995237)		(^d^ *u*_o_ = 1553.79)	
		0.05334	0.998704	110.69	1554.82	11.93
		0.10306	1.001917	110.60	1555.43	11.98
		0.11587	1.002748	110.46	1555.57	12.02
		0.16129	1.005659	110.04	1556.11	12.06
		0.28817	1.013661	109.48	1557.60	12.20
		0.31479	1.015365	109.21	1557.83	12.24
		0.50949	1.027427	108.26	1559.75	12.45
			(^c^ *ρ*_o_ = 0.993184)		(^d^ *u*_o_ = 1559.98)	
318.15		0.05334	0.996620	111.64	1560.03	13.78
		0.10306	0.999815	111.37	1560.50	13.76
		0.11587	1.000644	111.23	1560.59	13.83
		0.16129	1.003549	111.03	1560.98	13.86
		0.28817	1.011567	110.62	1562.00	13.99
		0.31479	1.013285	110.37	1562.10	14.05
		0.50949	1.025399	109.68	1563.29	14.22
323.15			(^c^ *ρ*_o_ = 0.990963)		(^d^ *u*_o_ = 1564.84)	
		0.05334	0.994369	112.33	1565.43	15.43
		0.10306	0.997519	112.21	1565.79	15.48
		0.11587	0.998337	112.10	1565.87	15.47
		0.16129	1.001187	112.03	1566.19	15.52
		0.28817	1.009067	111.72	1567.01	15.63
		0.31479	1.010735	111.55	1567.11	15.67
		0.50949	1.022542	111.08	1568.14	15.84
328.15			(^c^ *ρ*_o_ = 0.988575)		(^d^ *u*_o_ = 1568.48)	
		0.05334	0.991952	113.00	1569.01	17.90
		0.10306	0.995078	112.86	1569.18	17.88
		0.11587	0.995879	112.83	1569.21	17.94
		0.16129	0.998707	112.73	1569.35	17.97
		0.28817	1.006483	112.54	1569.72	18.09
		0.31479	1.008114	112.43	1569.76	18.12
		0.50949	1.019731	112.06	1570.18	18.28
293.15	*0*.*25*		(^c^ *ρ*_o_ = 1.002344)		(^d^ *u*_o_ = 1522.61)	
		0.05459	1.006053	107.69	1524.90	-0.37
		0.08555	1.008156	107.47	1525.83	-0.28
		0.12314	1.010692	107.35	1526.97	-0.25
		0.14154	1.011952	107.13	1527.48	-0.20
		0.19493	1.015563	106.81	1529.00	-0.10
		0.23030	1.017941	106.65	1530.00	-0.05
		0.24087	1.018665	106.54	1530.27	-0.02
			(^c^ *ρ*_o_ = 1.001089)		(^d^ *u*_o_ = 1534.27)	
298.15		0.05459	1.004751	108.60	1536.29	3.56
		0.08555	1.006821	108.46	1537.06	3.60
		0.12314	1.009322	108.33	1537.98	3.68
		0.14154	1.010554	108.19	1538.41	3.71
		0.19493	1.014097	107.95	1539.67	3.77
		0.23030	1.016441	107.77	1540.47	3.83
		0.24087	1.017159	107.64	1540.68	3.85
303.15			(^c^ *ρ*_o_ = 0.999601)		(^d^ *u*_o_ = 1544.44)	
		0.05459	1.003222	109.42	1546.25	7.34
		0.08555	1.005269	109.27	1546.84	7.37
		0.12314	1.007744	109.13	1547.54	7.43
		0.14154	1.008962	108.99	1547.86	7.47
		0.19493	1.012472	108.72	1548.79	7.55
		0.23030	1.014796	108.52	1549.37	7.61
		0.24087	1.015513	108.36	1549.50	7.64
			(^c^ *ρ*_o_ = 0.997905)		(^d^ *u*_o_ = 1553.07)	
308.15		0.05459	1.001488	110.20	1554.83	9.47
		0.08555	1.003517	110.01	1555.31	9.56
		0.12314	1.005968	109.86	1555.89	9.61
		0.14154	1.007172	109.73	1556.15	9.66
		0.19493	1.010661	109.39	1556.88	9.75
		0.23030	1.012959	109.21	1557.35	9.81
		0.24087	1.013651	109.13	1557.46	9.87
			(^b^ *ρ*_o_ = 0.996018)		(^d^ *u*_o_ = 1560.26)	
313.15		0.05459	0.999566	110.93	1561.55	13.81
		0.08555	1.001572	110.78	1561.81	13.87
		0.12314	1.004002	110.59	1562.10	13.96
		0.14154	1.005195	110.46	1562.23	13.99
		0.19493	1.008649	110.12	1562.57	14.08
		0.23030	1.010925	109.94	1562.79	14.11
		0.24087	1.011611	109.86	1562.84	14.13
			(^c^ *ρ*_o_ = 0.993950)		(^d^ *u*_o_ = 1566.07)	
318.15		0.05459	0.997456	111.79	1566.87	15.38
		0.08555	0.999442	111.61	1567.05	15.51
		0.12314	1.001839	111.47	1567.27	15.58
		0.14154	1.003008	111.41	1567.38	15.59
		0.19493	1.006393	111.20	1567.64	15.71
		0.23030	1.008621	111.08	1567.82	15.74
		0.24087	1.009296	111.00	1567.84	15.78
			(^c^ *ρ*_o_ = 0.991716)		(^d^ *u*_o_ = 1570.28)	
		0.05459	0.995197	112.37	1570.39	17.18
		0.08555	0.997174	112.11	1570.49	17.20
		0.12314	0.999571	111.86	1570.58	17.28
		0.14154	1.000745	111.72	1570.62	17.29
		0.19493	1.004134	111.43	1570.70	17.39
		0.23030	1.006372	111.25	1570.75	17.41
		0.24087	1.007051	111.15	1570.74	17.42
			(^c^ *ρ*_o_ = 0.989322)		(^d^ *u*_o_ = 1573.88)	
		0.05459	0.992777	112.98	1574.02	19.32
		0.08555	0.994736	112.75	1574.01	19.37
		0.12314	0.997104	112.58	1573.98	19.46
		0.14154	0.998280	112.35	1573.92	19.53
		0.19493	1.001631	112.12	1573.83	19.61
		0.23030	1.003862	111.88	1573.71	19.69
		0.24087	1.004547	111.73	1573.64	19.71

^a^ *m*_B_ is the molality of [DEEA][Pro] in water.

^b^ *m* is the molality of ascorbic acid in water or water + [DEEA][Pro] solutions.

^c^ *ρ*_o_ is the density of [DEEA][Pro] in water.

^d^ *u*_o_ is the speed of sound of [DEEA][Pro] in water.

The standard uncertainties are *u* (*T*) = 0.01 K, *u*(*m*) = 1.03·10^−5^ mol·kg^−1^, *u*(*ρ*) = 7.0·10^−3^ kg·m^−3^, *u* (*u*) = 0.5 m·s^−1^, *u* (*P*) = 0.05 kPa. The combined uncertainties, *U* are *U* (*V*_ϕ_) = (0.20 to 0.04)·10^6^ m^3^·mol^-1^ and *U* (*K*_*s2*,ϕ_) = (0.60 to 0.12)·10^−15^ m^3^·mol^-1^·Pa^-1^ for low and high concentration range of ascorbic acid, respectively (level of confidence, *k* = 0.95). The experiment was conducted under atmospheric pressure.

The experimentally measured densities and speeds of sound of the solutions were used to evaluate the isentropic compressibility, *κ*_s_ as:*κ*_s_ = *u* ^-2^.*ρ* ^-1^. The combined uncertainties in *V*_2, ϕ_values resulting from experimentally measured quantities[*u*(*m*) = 1.03·10^-5^ mol·kg^-1^, *u*(*ρ*) = 7.0·10^−3^kg·m^−3^,*u* (*T*) = 0.01 K]ranges from (0.20 to 0.04). 10^6^ m^3^·mol^-1^at (≤ 0.05 mol·kg^-1^) and high concentration range of AA, respectively and combined uncertainties in *K*_s,2, ϕ_ values ranges from(0.60 to 0.12)·10^−15^m^3^·mol^-1^·Pa^-1^at low and high concentration range of AA, respectively (level of confidence, *k* = 0.95).

**Fig 1:**
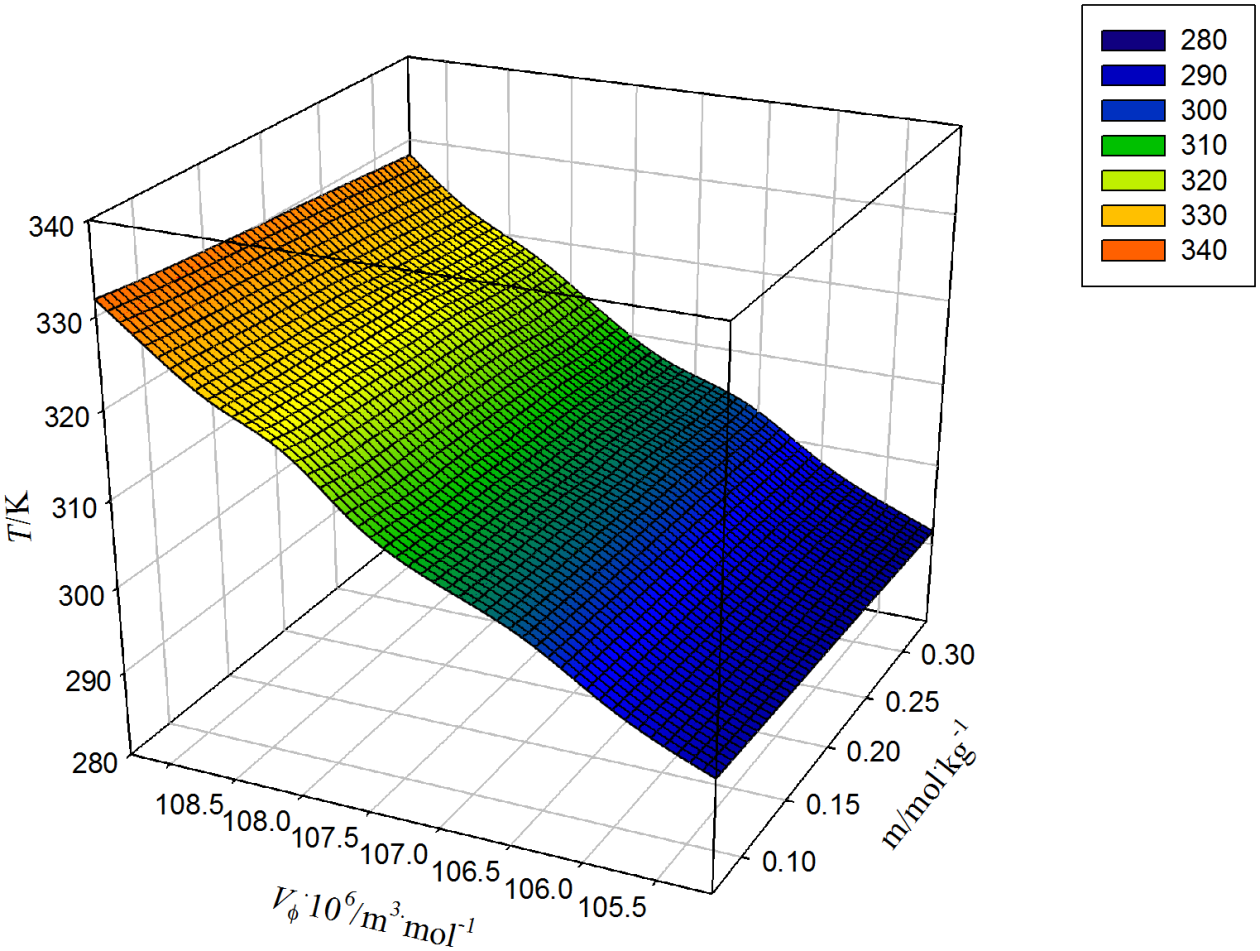
Plot of apparent molar volumes, *V*_ϕ_versus molalities, *m* of ascorbic acid in water at temperatures, *T* = (293.15, 298.15, 303.15, 308.15, 313.15, 318.15, 323.15 and 328.15) K.

The *V*_2,ϕ_and *K*_s,2,ϕ_values (Table 1) of AAin water increase with increase in concentration of AA and temperature, whereas in aqueous [DEEA][Pro] solutions *V*_2,ϕ_ values decrease with increase in concentration of AA. The variation of *V*_2,ϕ_versus molality of AA in water at different temperature is shown in Fig 1. In this plot, *V*_2,ϕ_ value increase as color of the figure change from dark blue region to dark orange region. The*K*_s,2,ϕ_value of AA in water and in aqueous solutions of [DEEA][Pro] are negative as well positive (at high temperature and cosolute concentration). For ionic compounds in water, highly negative *K*_s,2,ϕ_values have been observed, whereas for hydrophobic solute*K*_s,2,ϕ_values are positive and for uncharged hydrophilic solutes,*K*_s,2,ϕ_values are intermediate (small and negative). Negative *K*_s,2,ϕ_values suggest that, AA gets hydrated due to electrostricted water molecules in the vicinity of hydrophilic groups of AA. At high temperature and cosolute ([DEEA][Pro]) concentration, *K*_s,2,ϕ_values increase and become positive, which indicate the removal of electrostricted water around the solute (AA) molecules. Due to the presence of both hydrophilic and hydrophobic groups in AA, the *K*_s,2,ϕ_values obtained reflects the competition between various types of interactions between AA and solvent [32].

### 3.2 Infinite dilution partial molar volume and partial molar isentropic compression

Infinite dilution partial molar volume(*V*_2_° = *V*_2_°_,ϕ_)and partial molar isentropic compression(*K* °_s,2_ = *K*°_s,2,ϕ_) has been evaluated by least-square fitting to the corresponding data as Eqs (3) and (4):

$$V_{2,\phi }=\;V_{2}^{\text{o}}\;+\;S_{V}m$$ (3)

$$K_{\text{s},2,\phi }=\;K_{\text{s},2}^{\text{o}}\;+\;S_{K}m$$ (4)

where *S*_v_ and *S*_K_are respective experimental slopes. Apparent molar volume, *V*_2,ϕ_ of AA in water has been reported by various workers [33, 49–52] and in few reports,*V*_2,ϕ_ values were determined without considering the ionization of AA. Ayranci et al. [33] studied the volumetric properties of AA in 0.01 M HCl solutions, by suppressing the ionization of AA. A modified Debye-Huckel equation was proposed by Hakin, Mudrack, &Beswick [49] to consider the partial dissociation of AA in water, since at high concentration of weak acid, degree of hydrolysis is small, later some workers reported the volumetric properties of AA in water [50–52] without considering the dissociation of AA. *Dhondgeet al*. [40] *have* reported that at low concentration the effect of hydrolysis of AA on volumetric properties are assumed to be negligible (as it lies in the range of uncertainties), whereas at high concentration it is smaller due to decrease in the degree of hydrolysis. In this work, *V*_2,ϕ_ values of AA were determined by assuming that the effect of hydrolysis of AA on apparent molar volume is very small and negligible [40]. The *V*_2_° and *K* °_s,2_values of AA in water agree well the literature values [32,29,33,49–50,52] and are given in Tables 2 and 3, respectively. The *V*_2_°values of AA increase with concentration of [DEEA][Pro] and temperature, which indicate an increase in interactions between ions of [DEEA][Pro] (−NH_3_^+^, C_2_H_5_COO^−^) and AA, which may be due to the dominance of hydrophilic-ionic interactions. Both negative and positive *K*°_s,2_values were observed for AA in water and also in presence of [DEEA][Pro],which increase with concentration of PIL and temperature, thus indicating reduction in the electrostriction.

**Table 2:** Infinite dilution partial molar volumes, *V*_2_ºof ascorbic acid in water and in aqueous solutions of [DEEA][Pro]at *T* = (293.15 to 328.15) K.

^a^ *m*_B_/mol·kg^−1^	*V*_2_ °·10^6^/m^3^·mol^−1^							
*T*/K =	293.15	298.15	303.15	308.15	313.15	318.15	323.15	328.15
0.00	105.07±0.01^b^	105.76±0.01	106.25±0.01	106.94±0.01	107.51±0.01	107.86±0.01	108.44±0.01	108.93±0.01
	(0.85)^c^	(0.81)	(0.83)	(1.21)	(0.99)	(1.36)	(1.40)	(1.13)
		[105.95^d^,103.46^e^,106.49^f^,105.17^g^,105.40^h^ ^,^ ^I^,105.38^j^]		[106.98^d^,104.98^e^,107.08^f^,106.97^g^]		[107.94^d^]		
0.10	107.03±0.03 (-3.95)	108.05±0.04 (-4.03)	108.76±0.04 (-4.93)	109.50±0.07 (-6.87)	110.25±0.08 (-6.01)	110.75±0.06 (-6.50)	111.45±0.06 (-5.97)	112.03±0.07 (-6.31)
0.15	107.00±0.06 (-3.53)	107.89±0.04 (-2.60)	108.72±0.06 (-2.46)	109.53±0.04 (-2.31)	110.30±0.04 (-2.61)	110.02±0.05 (-1.98)	111.68±0.04 (-2.28)	112.33±0.03 (-2.54)
0.20	107.90±0.08 (-4.66)	108.71±0.05 (-3.81)	109.55±0.06 (-3.92)	110.33±0.06 (-4.66)	111.07±0.09 (-5.81)	111.77±0.08 (-4.19)	112.46±0.04 (-2.74)	113.08±0.03 (-1.99)
0.25	108.02±0.04 (-6.09)	108.90±0.04 (-5.01)	109.75±0.05 (-5.48)	110.52±0.03 (-5.74)	111.27±0.02 (-5.83)	111.98±0.02 (-4.01)	112.66±0.03 (-6.29)	113.32±0.05 (-6.39)

^a^ *m*_B_, molality of [DEEA][Pro] in water.

^b^standard deviation.

^c^S_v_ /m^3^·kg·mol^−2^.

^d^Ref [32].

^e^Ref [33].

^f^Ref [49].

^g^Ref [50].

^h^Ref [52].

^i^Ref [29].

**Table 3:** Infinite dilutionpartial molar isentropic compression, *K* °_s_,2 of ascorbic acid in water and in aqueous solutions of [DEEA][Pro]at *T* = (293.15 to 328.15) K.

^a^ *m*_B_/mol·kg^−1^	_*K*_°_s,2_· 10^−15^ / m^3^·mol^−1^· Pa^−1^							
*T*/K =	293.15	298.15	303.15	308.15	313.15	318.15	323.15	328.15
Ascorbic acid								
0.00	-12.25±0.01^b^	-8.68±0.02	-5.60±0.02	-4.08±0.01	-0.08±0.01	1.03±0.02	2.48±0.01	3.97±0.02
	(1.23)^c^	(1.38)	(1.19)	(1.21)	(1.11)	(1.38)	(1.26)	(1.22)
		[-8.04^d^,-7.10^e^,-9.00^f^]		[-4.06^d^,-4.00^c^]		[0.99^d^]		
0.10	-3.64±0.02 (1.27)	1.04±0.01 (1.18)	4.50±0.04 (1.16)	6.14±0.01 (1.21)	10.48±0.01 (1.69)	12.73±0.03 (1.38)	14.36±0.01 (1.04)	16.11±0.02 (1.06)
0.15	-2.20±0.01 (0.83)	1.85±0.03 (0.91)	5.27±0.01 (0.80)	7.07±0.02 (0.78)	11.38±0.01 (0.81)	13.48±0.01 (0.93)	15.08±0.01 (0.72)	17.25±0.02 (0.80)
0.20	-1.81±0.01 (1.08)	2.18±0.02 (1.01)	5.80±0.02 (1.04)	7.68±0.01 (0.97)	11.87±0.01 (1.14)	13.70±0.03 (1.04)	15.38±0.01 (0.91)	17.83±0.02 (0.90)
0.25	-0.46±0.01 (1.80)	3.48±0.01 (1.54)	7.24±0.01 (1.62)	9.37±0.02 (1.98)	13.73±0.02 (1.69)	15.32±0.03 (1.94)	17.10±0.01 (1.37)	19.20±0.01 (2.12)

^a^ *m*_B_, molality of [DEEA][Pro] in water.

^b^standard deviation.

^c^S_v_ /m^3^·kg·mol^−2^·Pa^-1^.

^d^Ref [32].

^e^Ref [52].

^f^Ref [29].

**Fig 2:**
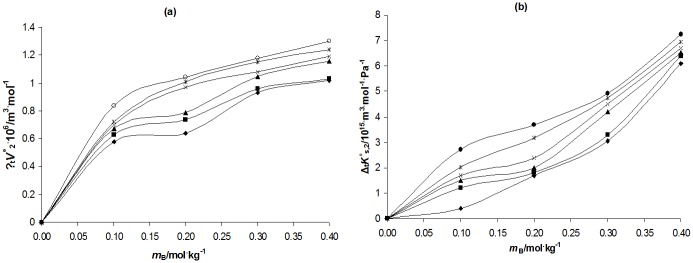
Plots of volumes of transfer(Δ_t_ *V*_2_°and Δ_t_ *K°*_s,2_) versus molalities, *m*_B_ of [DEEA][Pro] of ascorbic acid. a) ΔtV_2_°versus *m*_B_; b) Δt*K*°s,_2_ versus *m*_B_ at temperatures, T = ♦, 293.15 K; ■, 298.15 K; ▲,303.15 K; ×, 308.15 K, *, 313.15 K, ☼, 318.15 K, +, 323.15 K,-, 328.15 K.

**Fig 3:**
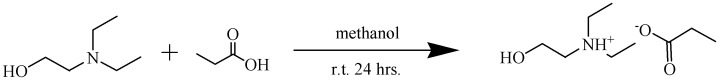
Synthesis of diethylethanolammonium propanoate [DEEA][Pro].

The influence of PIL on solvation behaviour of AA can be studied on the basis of partial molar volumes of transfer, (Δ_t_ *V*_2_°and Δ_t_ *K*°_s,2_), which is considered to be free from solute-solute interactions, and werecalculated using Eq (5):

$$\Delta_{t}X_{2}^{o}\;\;=\;\;X_{2\;}^{\text{o}}\;\left(\text{in aqueous }\left[\text{DEEA}\right]\left[\text{Pro}\right]\text{solutions}\right)\;\;\;-\;\;\;\;X_{2\;}^{\text{o}}\;\left(\text{in water}\right)$$ (5)

where Δ_t_ *X*_2_° = (Δ_t_ *V*_2_° or Δ_t_ *K*°_s,2_), *X*_2_° = (*V*_2_° or *K*°_s,2_), the plot of Δ_t_ *V*_2_°versus *m*_B_ has been illustrated in Fig 2a and Δ_t_ *K* °_s,2_ versus *m*_B_ in Fig 2b. The transfer parameters (Δ_t_ *V*_2_°and Δ_t_ *K*°_s,2_) are positive and increase with concentration of newly synthesized PIL (synthesis is shown in Fig 3) and temperature. The Δ_t_ *V*_2_° values increase with cosolute (PIL) concentration, however a slight decrease in Δ_t_ *V*_2_° values from *m*_B_ ≈ (0.10 to 0.15) mol·kg^−1^ have been observed, whereas Δ_t_ *K*°_s,2_ values increase continuously with PIL concentration at all temperature. This behaviour in Δ_t_ *V*_2_° values for AA is different (only between [0.10 to 0.15] mol·kg^−1^) as compared to Δ_t_ *K*°_s,2_ values. The observed difference may be due to the fact that apparent molar isentropic compression is more sensitive parameter as compared to apparent molar volume [44] in measuring the structural changes occurring in solutions.

**Fig 4:**
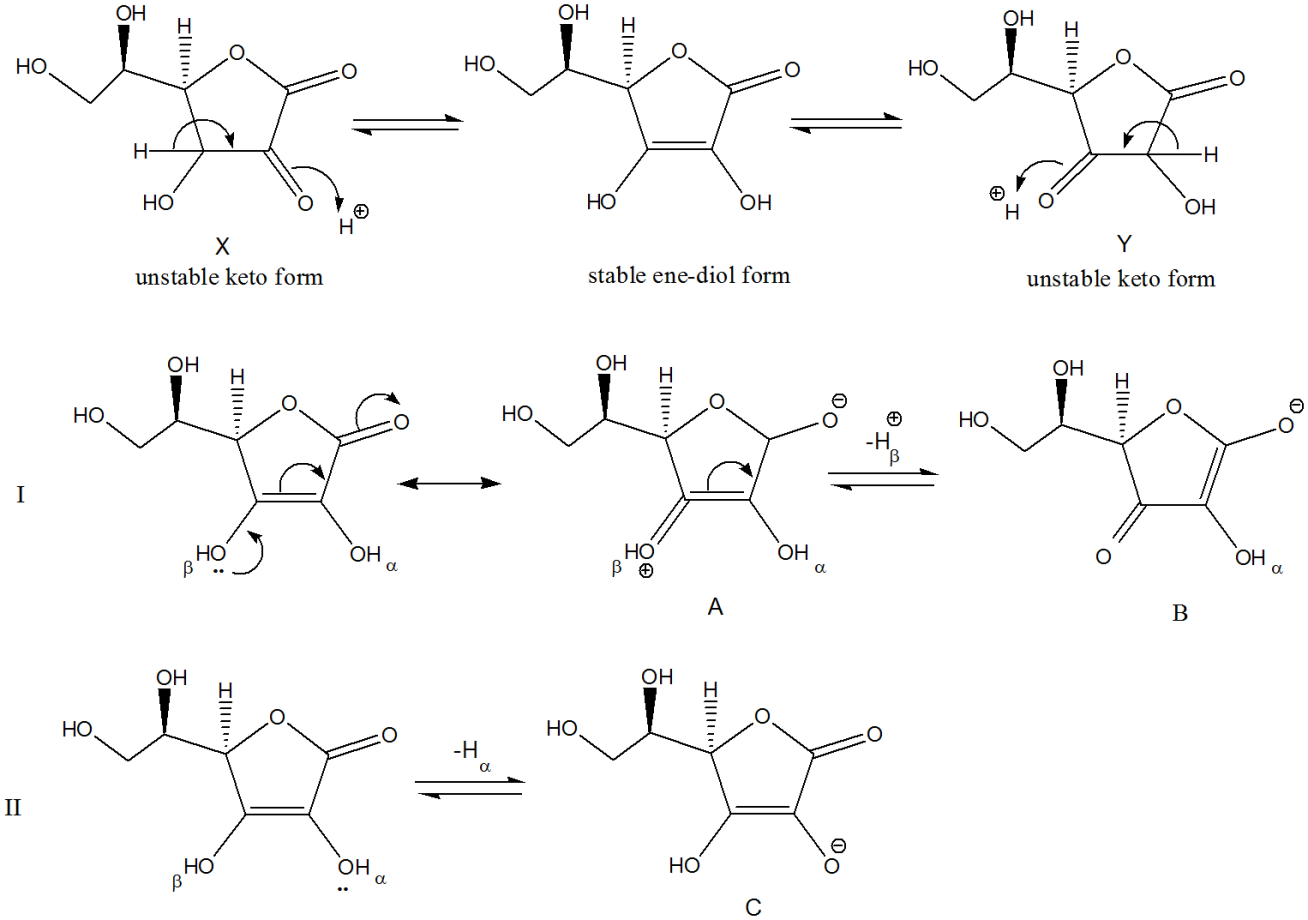
1,2 Diketone (X) and 1,3-diketone (Y) form of ascorbic acid and their interconversion.

Ascorbic acid has a five membered ring containing two carbonyl groups and exists as [53] conjugate ene-diol (having two unstable keto form and one stable ene-diol form) as shown in Fig 4. The enol form is stable and is delocalized, as shown in Fig 4.

The two possible forms of AAin solutions are: 1,2 diketone (X) and 1,3-diketone (Y) as shown in Fig 4, and these forms rapidly interconvert (Fig 4). AA contains two acidic protons, namely, H_α_ and H_β_(I of Fig 4), on dissociation of any of these protons, ascorbate ion is formed. The stability of respective ascorbate ion formed determines the acidity of proton (H_α_ or H_β_). As shown in Fig 4, structure A has one more equally contributing resonating structure B [53], therefore the stability of conjugate base on removal of H_β_ is more (shown in I of Fig 4) as compared to conjugate base generated on removal of H_α_ (shown in II of Fig 4). The carboxylate anion of [DEEA][Pro] will probably interact more strongly with H_β_ (most acidic hydrogen), which may be responsible for positive transfer volumes (Δ_t_ *V*_2_° or Δ_t_ *K*°_s,2_), further, it has been observed that these interactions dominate over the whole concentration range of PIL. Aryanci et al. [33] studied the solvation behaviour of AA in aqueous sodium chloride solutions (in 0.01 M HCl) and they observed no regular trend in Δ_t_ *V*_2_° values for AA, and suggested that the irregular trend in Δ_t_ *V*_2_° values indicate the presence of complex interactions between solute and cosolute in aqueous NaCl solutions (containing background 0.01 M HCl). However, in the present study, regular trend observed in Δ_t_ *V*_2_° and Δ_t_ *K*°_s,2_ values suggest that with increase in PIL concentration and temperature, the hydrophilic-ionic types of interactions become more favorable.

The Δ_t_ *V*_2_° and Δ_t_ *K*°_s,2_values can be interpreted in terms of structural interaction model [54] and group additivity model [55]. According to these models [54–55], the type of interactions between AA and [DEEA][Pro] in ternary solutions can be classified as: a)Hydrophobic—cation interactions between hydrophobic parts of AA and—NH_3_^+^ of PIL; b) Hydrophilic—cation interactions between (–OH,—C = O, and—O–) groups of AA and −NH_3_^+^; c) Hydrophobic—anion interactions between hydrophobic parts of AA and C_2_H_5_COO^−^; d) Hydrophilic—hydrophobic interactions between the hydrophilic groups of AA and hydrophobic parts of [DEEA][Pro]. According to the structural interaction model [54], type(a, c and d)interactions are repulsive as the groups involved are incompatible or inability of the groups to orient water, contribute negative volume. Interactions of type b contribute positive to transfer volume due to the overlap of hydration co-sphere of ions of [DEEA][Pro] and hydrophilic groups (–OH,—C = O, and—O–) of AA, which leads to a decrease in structure-breaking tendency of ion and thus reduction of electrostriction. Transfer volumes (Δ_t_ *V*_2_° and Δ_t_ *K*°_s,2_) increase with cosolute concentration and temperature, which indicate dominance of hydrophilic-ionic interactions (type b) over hydrophobic type of interactions. The positive Δ_t_ *V*_2_° values of AA in presence of NaCl [33] and in PEG3350[29] have also been reported,which also suggests the dominance of hydrophilic type of interactions.

Thethermal expansion coefficients (∂*V*_2_°/∂*T*)_P_ and its second derivatives (∂^2^ *V*_2_°/∂*T* ^*2*^)_P_have been calculated by using the following Eq (6):

$$V_{2}^{o}\;\;=\;\;v_{o}+\;v_{1}T+v_{2}T^{2}$$ (6)

where *ν*_o_, *ν*_1_ and *ν*_2_ are constants. The derivative of *V*_2_° with respect to temperature at constant pressure i.e. (∂*V*_2_°/∂*T*)_P_for AA in water and in presence of [DEEA][Pro] is given in Table 4. Hepler [56] used a mathematical equation, to deduce information regarding structure-making or-breaking ability of an ion in solution. According to Hepler’s [56] criteria: ( δCP,2oδP) T = -T ∂ 2V2o∂T2 P  , negative (∂^2^ *V*_2_°/∂*T* ^*2*^)_P_ values obtained for AA in water and in aqueous PIL solutions suggest that AA behave as structure breaker (chaotropes). Banipal et al.[32] and Dhonge et. al. [40] have also reported that AA behaves as structure breaker in water.

**Table 4:** Partial molar expansion coefficients, *(*∂*V2∞/*∂*T)*_P_ and *(*∂^*2*^ *V2∞/*∂*T* ^*2*^ *)*_P_ of ascorbic acid) in water and in aqueous [DEEA][Pro] solutions at temperatures, *T* = (293.15 to 328.15) K.

*m*_B_ /mol·kg^−1^	*(∂V*_*2*_^*∞*^ */∂T)*_P_·10^6^								SD*	*(∂* ^*2*^ *V*_*2*_^*∞*^ */∂T* ^*2*^ *)*_P_·10^6^
	m^3^·mol^−1^·K^−1^									m^3^·mol^−1^·K^−2^
	*T*/K									
	293.15	298.15	303.15	308.15	313.15	318.15	323.15	328.15		
0.00	0.129	0.123	0.118	0.112	0.106	0.101	0.095	0.089	0.07	-0.001
0.10	0.176	0.166	0.155	0.145	0.135	0.124	0.114	0.104	0.08	-0.002
0.15	0.181	0.173	0.165	0.156	0.148	0.140	0.131	0.123	0.01	-0.002
0.20	0.173	0.166	0.159	0.152	0.145	0.138	0.131	0.124	0.01	-0.001
0.25	0.177	0.170	0.162	0.155	0.147	0.139	0.132	0.124	0.02	-0.002

*SD—standard deviation.

### 3.3 Interaction coefficients

Pair (*Y*_AB_) and triplet (*Y*_ABB_) volumetric and compressioninteraction coefficients have been calculated from corresponding volume of transfer (Δ_t_ *V*_2_° or Δ_t_ *K*°_s,2_) based on McMillan-Mayer theory [57] of solutionsby using Eq (7):

$$\Delta_{t}Y_{2}^{o}\;\;=\;2Y_{AB}m_{B}\;+\;3Y_{ABB}m_{B}^{2}$$ (7)

where A denotes AA and B denotes PIL ([DEEA][Pro]). Constants *Y*_AB_(*V*_AB_ or *K*_AB_) and *Y*_ABB_(*V*_ABB_ or*K*_ABB_) are pair and triplet volumetric or compressioninteraction coefficients, respectively. Pair interaction coefficients, *Y*_AB_ contribute positively and triplet coefficients, *Y*_ABB_can contribute negatively (Tables 5 and 6). Pair volumetric interaction coefficients, *Y*_AB_are found to be positive and triplet interaction coefficients, *Y*_ABB_ are negative, at all temperatures. Overall, triplet volumetric interaction coefficients, *Y*_ABB_ are small, which indicate that the interactions between AA and [DEEA][Pro] are mainly pair wise. Positive values of both *V*_AB_ and *K*_AB_ parameters suggest that interactions occur due to the overlap of hydration co-spheres of AA and ions of PIL.

**Table 5:** The pair,*V*_AB_ and triplet,*V*_ABB_ interaction coefficients for ascorbic acid in aqueous[DEEA][Pro]solutions at *T* = (293.15 to 328.15)K.

*V*_AB_·10^6^	*V*_ABB_·10^6^	*V*_AB_·10^6^	*V*_ABB_·10^6^
m^3^·mol^-2^·kg	m^3^·mol^-3^·kg^2^	m^3^·mol^-2^·kg	m^3^·mol^-3^·kg^2^
*T* = 293.15 K		*T* = 298.15 K	
10.18	−11.42	11.84	−15.16
*T* = 303.15 K		*T* = 308.15 K	
13.37	−17.32	13.89	−18.25
*T* = 313.15 K		*T* = 318.15 K	
15.04	−20.42	16.39	−22.04
*T* = 323.15 K		*T* = 328.15 K	
17.02	−23.22	17.65	−24.01

**Table 6:** The pair, *K*_AB_ and triplet, *K*_ABB_ compressibility interaction coefficients for ascorbic acid in aqueous[DEEA][Pro] solutions at temperatures, *T* = (293.15 to 328.15) K.

*K*_AB_	*K*_ABB_	*K*_AB_	*K*_ABB_
m^3^·mol^−2^·Pa^−1^·kg	m^3^·mol^−3^·Pa^−1^·kg^2^	m^3^·mol^−2^·Pa^−1^·kg	m^3^·mol^−3^·Pa^−1^·kg^2^
*T* = 293.15 K		*T* = 298.15 K	
50.86	−75.33	56.52	−89.24
*T* = 303.15 K		*T* = 308.15 K	
58.07	−89.86	58.33	−87.39
*T* = 313.15 K		*T* = 318.15 K	
60.05	−90.42	67.68	−108.69
*T* = 323.15 K		*T* = 328.15 K	
68.33	−108.75	71.29	−112.65

## Conclusions

The volumetric and compressiontransfer volumes (Δ_t_ *V*_2_° and Δ_t_ *K*°_s,2_) for AA in presence of diethylethanolammonium propionate ([DEEA][Pro]) were found to be positive, which increase with increase in concentration of [DEEA][Pro] and temperature. The positive Δ_t_ *V*_2_° and Δ_t_ *K*°_s,2_values suggest the dominance of hydrophilic-ionic type of interactions between the ions of PIL and hydrophilic sites of AA (−OH, −C = O, −O−), due to the overlap of hydration co-sphere of PIL and AA.

The negative values of second derivative i.e. (∂^2^ *V*_2_°/∂*T* ^*2*^)_P_ obtained for AA in water and also in aqueous solutions of [DEEA][Pro] suggest that AA behave as structure breaker. The values of pair interaction coefficients (*V*_AB_ and *K*_AB_) were found to be positive, which further support the view that interactions occur due to the overlap of hydration co-sphere of AA and ions of PIL. The transfer parameters and interaction coefficients suggest that AA interacts strongly with [DEEA][Pro] in aqueous solution.
